# The Antagonism of Law

**Published:** 1897-09

**Authors:** 


### THE ANTAGONISM OF LAW.

The ill-judged proceedings of some connected with the administration of dental laws has created a wide-spread feeling of disquiet in college circles, and has led, in repeated instances, to open antagonism. This is to be deplored, for it necessarily unsettles educational processes and creates inharmony where unity of sentiment and action should alone prevail.

The National Association of Dental Faculties has, throughout this trying period in dental education, aimed to conserve all interests, and has honestly endeavored to establish fraternal relations with the National Association of Dental Examiners, in the hope that time would eventually quiet all disturbing elements and permit the two bodies to work together for the advancement of educational methods. With this idea a joint committee was proposed by this body, at its recent meeting at Old Point Comfort, the purpose of which was to propose a plan for the settlement of all differences and arrange a basis of work upon which both Associations could rest. This proposition was acceded to by the National Association of Dental Examiners, and the two committees met in joint session. The indications, at first, all pointed to a harmonious settlement, and the general feeling, on the part of the members of the National Association of Dental Faculties, was one of gratification that they had heard the last of these criminations and recriminations, so frequent of recent years.

This peaceful state of things was, it seems, altogether illusory, for at the very last hour of the session of the Examiners there was adopted a series of rules which reaffirmed all the objectionable features previously formulated by the committee on schools of that body. This document was not sent officially to the National Association of Dental Faculties, hence criticism, in detail, is deferred until the report of proceedings is at hand; but as there is no question as to the fact that such rules were adopted and subsequently read at the final meeting of the Faculties, it is not improper to state here that this attempt to control the curriculum of each of the dental colleges of this country will be resisted and treated with the contempt it deserves, and, further, that the threat which is supposed to give force to the rules that colleges refusing to submit will be severely dealt with (the substance not the words being given) will utterly fail of its hoped-for effect, for should any State board attempt to carry this rule into effect, it would very speedily find itself outside of the pale of the law and could legally accomplish nothing.

The claim has been made that the National Association of Dental Examiners has been working solely for the good of the dental profession, and it is freely and gratefully acknowledged that with many active therein this is true, but this cannot be said of all. The action of some in antagonizing colleges and holding them up to unjust ridicule, evidences the fact that the spirit manifested is not one leading to harmony, but to that of injury, if not to the eventual destruction of all dental laws in the United States.

The action of this National Association of Dental Examiners, being without legal force and without the concurrence of the entire number of State boards, must fail in the effect desired, and it is to be hoped that the dental colleges of the country will quietly continue their work regardless of this action, leaving the question to be settled, if settled at all, by the higher courts.

